# The severity and risk factors for mortality in immunocompromised adult patients hospitalized with influenza-related pneumonia

**DOI:** 10.1186/s12941-021-00462-7

**Published:** 2021-08-24

**Authors:** Liang Chen, Xiudi Han, YanLi Li, Chunxiao Zhang, Xiqian Xing

**Affiliations:** 1 Department of Infectious Diseases, Nanjing Lishui People’s Hospital, Lishui District, No. 86 Chongwen Road, Nanjing, China; 2 Department of Infectious Diseases, Beijing Jishuitan Hospital, 4Th Medical College of Peking University, Beijing, China; 3 Department of Pulmonary and Critical Care Medicine, Qingdao Municipal Hospital, Qingdao City, Shandong Province China; 4 Department of Infectious Diseases and Clinical Microbiology, Beijing Chao-Yang Hospital, Capital Medical University, Beijing, China; 5 Department of Pulmonary and Critical Care Medicine, Beijing Huimin Hospital, Beijing, China; 6 Department of Pulmonary and Critical Care Medicine, The 2Nd People’s Hospital of Yunnan Province, Kunming City, Yunnan Province China

**Keywords:** Immunocompromised, Influenza-related pneumonia, Severity, Risk factor, Mortality

## Abstract

**Objective:**

To explore disease severity and risk factors for 30-day mortality of adult immunocompromised (IC) patients hospitalized with influenza-related pneumonia (Flu-p).

**Method:**

A total of 122 IC and 1191 immunocompetent patients hospitalized with Flu-p from January 2012 to December 2018 were recruited retrospectively from five teaching hospitals in China.

**Results:**

After controlling for confounders, multivariate logistic regression analysis showed that immunosuppression was associated with increased risks for invasive ventilation [*odds ratio:* (*OR*) 2.475, *95% confidence interval* (*CI*): 1.511–4.053, p < 0.001], admittance to the intensive care unit (*OR:* 3.247, *95% CI* 2.064–5.106, p < 0.001), and 30-day mortality (*OR:* 3.206*, 95% CI* 1.926–5.335, p < 0.001) in patients with Flu-p. Another multivariate logistic regression model revealed that baseline lymphocyte counts (*OR:* 0.993*, 95% CI* 0.990–0.996, p < 0.001), coinfection (*OR:* 5.450*, 95% CI* 1.638–18.167, p = 0.006), early neuraminidase inhibitor therapy (*OR* 0.401*, 95% CI* 0.127–0.878, p = 0.001), and systemic corticosteroid use at admission (*OR:* 6.414*, 95% CI* 1.348–30.512, p = 0.020) were independently related to 30-day mortality in IC patients with Flu-p. Based on analysis of the receiver operating characteristic curve (ROC), the optimal cutoff for lymphocyte counts was 0.6 × 10^9^/L [area under the ROC (AUROC) = 0.824, 95% CI 0.744—0.887], sensitivity: 97.8%, specificity: 73.7%].

**Conclusions:**

IC conditions are associated with more severe outcomes in patients with Flu-p. The predictors for mortality that we identified may be valuable for the management of Flu-p among IC patients.

**Supplementary Information:**

The online version contains supplementary material available at 10.1186/s12941-021-00462-7.

## Introduction

Influenza is a common viral respiratory disease that affects between 5 and 10% of the world’s population each year, resulting in roughly 3–5 million severe infections and 290,000–650,000 annual deaths [1, 2]. Influenza-related pneumonia (Flu-p) is a severe form of influenza infection associated with over 50% of influenza-related hospitalizations [3, 4].

In recent years, immunocompromised (IC) status has become increasingly recognized as frequent comorbidity of influenza [5]. Observational studies have shown that the influenza vaccine is less effective in IC populations [6, 7]. Together with decreased host defenses, IC individuals have increased susceptibility to influenza virus infection, and have a greater risk of severe outcomes [8, 9]. However, most previous researchers have only focused on patients with specific subtypes of IC conditions [e.g., stem cell transplantation, human immunodeficiency virus/acquired immune deficiency syndrome (HIV/AIDS)], which limits the generalizability of the conclusions [5, 8–10]. Data on patients with influenza-related pneumonia (Flu-p) is particularly scarce. Additionally, prognostic factors among IC patients with influenza remain largely unknown.

Here, we conducted a multicenter, real-world study that aimed to (i) assess disease severity and outcomes of IC adult patients hospitalized with community-onset Flu-p, and (ii) to explore the baseline predictors for mortality in these patients.

## Methods

### Patient recruitment

Patients who had been tested for the influenza virus at five tertiary hospitals in China between January 1st, 2012 and December 31st, 2018 (Additional file 1: Material S1) were screened for eligibility. Patients with confirmed Flu-p were enrolled in this study. Patients were excluded if they: (i) were not classified as having community-onset pneumonia (i.e., were hospitalized within the last 28 days for immunocompetent patients and 90 days for immunosuppressed patients, with pneumonia onset ≥ 48 h after admission [11]), as the inclusion of non-community-onset pneumonia cases could have complicated result interpretation; and (ii) were < 14 years old.

### Study definitions

Patients with Flu-p were defined as individuals for whom polymerase chain reaction (PCR) analyses of respiratory specimens (including sputum, nasal/nasopharyngeal swabs, bronchial aspirates, and bronchoalveolar lavage fluid) were positive for influenza viral RNA, and for whom respiratory symptoms and chest radiographic findings were consistent with newly emergent chest infiltrates. IC patients included primary immune deficiency diseases, active malignancy, HIV infection with a CD4 T-lymphocyte count < 200 cells/mL or percentage < 14%, immunosuppressive therapy (Additional file 1: Material S2), solid organ transplantation, hematopoietic stem cell transplantation, splenectomy [12]. Patients who had early neuraminidase inhibitor (NAI) therapy were those who had been administered NAI agents within two days of symptom onset [13]. Patients who had systemic corticosteroid treatment were those who had been administered one or more systemic corticosteroid doses at the day of admission. Patients with community-acquired co-infecting respiratory pathogens were those who had pathogens detected via standard microbiological techniques (Additional file 1: Material S3) within 48 h of admission [11].

### Data collection

Data were extracted from patient medical records with standardized case report forms, and included demographic details, comorbidities (see Additional file 1: Material S2), baseline symptoms, vital signs, laboratory results, radiographic findings, community-acquired co-infecting respiratory pathogens, patient management, and outcome data [including NAI use, antibiotic use, systemic corticosteroid administration, noninvasive/invasive ventilation, admittance to the intensive care unit (ICU), complications during hospitalization and 30-day mortality]. Outcomes for those hospitalized for < 30 days were established through telephone follow-up.

### Statistical analysis

A Kolmogorov–Smirnov test was used to assess result normality, with normally and non-normally distributed variables presented as means ± standard deviation ($\overline{x}$ ± SD) and medians, respectively. Continuous variables were evaluated with Mann–Whitney *U* tests or Student’s t-tests, whereas categorical variables were assessed with Fisher’s exact test or chi-squared tests. A two-tailed *p* < 0.05 indicated statistical significance. SPSS 22.0 or MedCalc 19.0 were used for all statistical tests.

To evaluate the effects of IC status on clinical outcomes, we performed multivariate backward stepwise logistic regression after adjusting for age, sex, duration from illness onset to hospital admission, influenza virus type, comorbidities, pregnancy, obesity, smoking history, receipt of early NAI therapy, systemic corticosteroid use and coinfection with other pathogens. Those risk factors had previously been associated with clinical outcomes in patients with influenza [14].

IC Flu-p patients were divided into surviving and deceased groups according to survival status at 30 days post-admission, and baseline characteristics were compared between the two groups. To explore the risk factors for 30-day mortality in IC, Flu-p patients, variables with *p*-values < 0.1 in univariate analyses were entered into the multivariate backward stepwise logistic regression analysis. The optimal cut-off and predictive value of lymphocyte counts were assessed using the area under the receiver characteristics curve (AUROC) based upon Youden’s index.

## Results

### Screening process

**Fig. 1:**
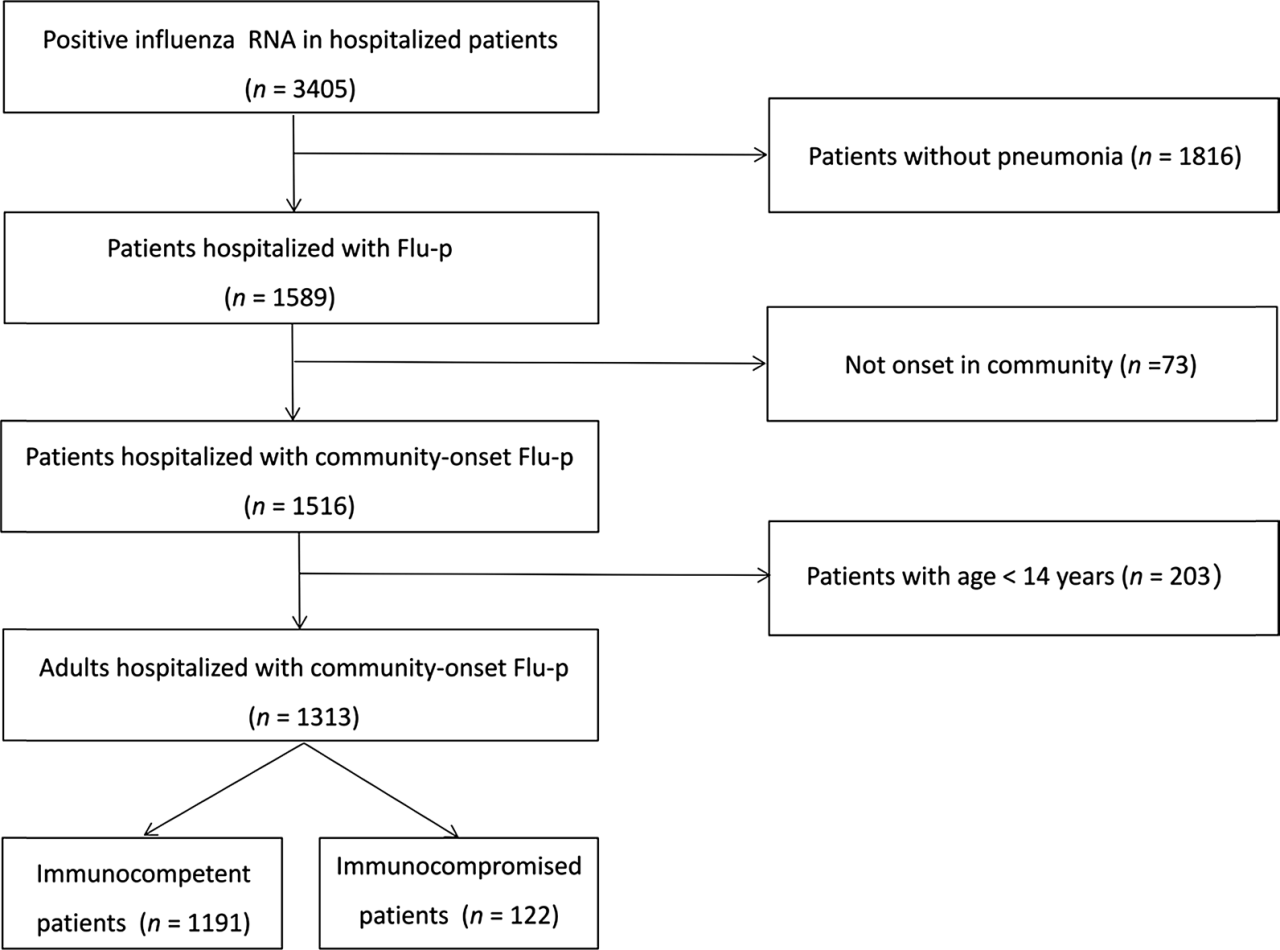
Screening algorithm of patients hospitalized with Flu-p. A total of 3405 hospitalized patients with RNA tests positive for influenza and 1313 eligible adult patients with Flu-p were included in the final analysis

A total of 3405 hospitalized patients with RNA tests positive for influenza and 1313 eligible adult patients with Flu-p were included in the final analysis, including 1191 immunocompetent patients and 122 IC patients (Fig. 1). The three most common immunocompromising conditions were immunosuppressive therapy (57/122, 46.7%), active malignancy (25/122, 20.5%) and organ transplantation (17/122, 13.9%) (Additional file 1: Material S4).

### Comparison of clinical characteristics and outcomes between IC and non-IC patients with Flu-p

Compared with non-IC patients, IC patients were younger (median: 45.0 years *vs.* 61.0 years, p < 0.001), and were admitted to hospitals earlier after illness onset (median: 2.0 days *vs.* 4.0 days, p < 0.001). Chest pain (31.1% *vs.* 17.2%, p < 0.001) and pleural effusion (47.5% *vs.* 32.0%, p < 0.001) at admission were present significantly more often in IC patients, while fever (63.9% *vs.* 76.6%, p < 0.001) was present significantly less often. The median baseline lymphocyte counts were significantly lower in IC patients than in non-IC patients (0.6 × 10^9^/L *vs.*1.0 × 10^9^/L, p < 0.001). Other baseline vital signs, symptoms, and laboratory and radiologic findings were similar between patients in the two groups (Table 1).

**Table 1:** The clinical characteristics and outcomes of IC and non-IC patients with Flu-p

Variable	Total (*n* = 1313)	IC (*n* = 122)	Non IC (*n* = 1191)	*p* value
Age (yrs, median, IQR)	59.0 (45.0–76.0)	45.0 (32.8–55.3)	61.0 (49.0–78.0)	** < 0.001**
Male (*n*,%)	710 (54.1)	61 (50.0)	649 (54.5)	0.343
Influenza A infection (*n*,%)	459 (35.0)	35 (28.7)	424 (35.6)	0.127
Days from illness onset to admission (median, IQR)	3.0 (2.0–5.0)	2.0 (1.0–3.0)	4.0 (2.0–5.0)	** < 0.001**
*Comorbidities (n,%)*				
Cardiovascular disease	309 (23.5)	22 (18.0)	287 (24.1)	0.133
Diabetes mellitus	162 (12.3)	14 (11.5)	148 (12.4)	0.761
Cerebrovascular disease	128 (9.7)	14 (11.5)	114 (9.6)	0.500
COPD	117 (8.9)	15 (12.3)	102 (8.6)	0.168
Chronic kidney disease	42 (3.2)	7 (5.7)	35 (2.9)	0.161
Asthma	41 (3.1)	5 (4.1)	36 (3.0)	0.706
Obesity (*n*,%)	86 (6.5)	5 (4.1)	81 (6.8)	0.250
Pregnancy (*n*,%)	9 (0.7)	0 (0.0)	9 (0.8)	1.000
Smoking history (*n*,%)	375 (28.6)	27 (22.1)	348 (29.2)	0.099
*Baseline clinical and radiologic features (n,%)*				
Fever ≥ 38℃	990 (75.4)	78 (63.9)	912 (76.6)	**0.002**
Myalgia	441 (33.6)	37 (30.3)	404 (33.9)	0.424
Sore throat	235 (17.9)	19 (15.6)	216 (18.1)	0.482
Cough	1290 (98.2)	119 (97.5)	1171 (98.3)	0.532
Sputum	1032 (78.6)	90 (73.8)	942 (79.1)	0.172
Chest pain	243 (18.5)	38 (31.1)	205 (17.2)	** < 0.001**
Respiratory rates ≥ 30 breaths/min	178 (13.6)	19 (15.6)	159 (13.4)	0.494
Altered mental status	179 (13.6)	12 (9.8)	167 (14.0)	0.199
SBP < 90 mmHg	21 (1.6)	4 (3.3)	17 (1.4)	0.121
Leukocytes (× 10^9^/L, median, IQR)	6.3 (5.2, 10.0)	6.3 (4.5, 10.1)	6.3 (5.2, 10.0)	0.390
Lymphocytes (× 10^9^/L, median, IQR)	08 (0.6, 1.6), *n* = 1211	0.6 (0.5, 1.0)	1.0 (0.6, 1.7), *n* = 1089	** < 0.001**
HB (g/L, mean ± SD)	123.1 ± 23.0	122.9 ± 23.2	123.1 ± 23.0	0.907
ALB (g/L, mean ± SD)	28.7 ± 5.4, *n* = 1169	28.4 ± 6.0	28.8 ± 5.3, *n* = 1047	0.451
BUN (mmol/L, median, IQR)	5.3 (3.6, 8.3), *n* = 1221	6.6 (3.1, 8.4)	5.1 (3.1, 8.4), *n* = 1099	0.348
PaO_2_/FiO_2_ (mmHg, median, IQR)	317.5 (249.0, 347.0), *n* = 1170	317.5 (266.3, 340.0)	316.2 (244.4, 458.6), *n* = 1048	0.678
Multilobar infiltrates	958 (73.0)	85 (69.7)	873 (73.3)	0.390
Pleural effusion	439 (33.4)	58 (47.5)	381 (32.0)	**0.001**
Coinfection (*n*, %)	458 (34.9)	53 (43.4)	405 (34.0)	**0.037**
*Clinical treatment and outcomes*				
Early NAI therapy (*n*,%)	495 (37.7)	55 (45.1)	437 (36.7)	0.068
Systemic corticosteroids use at admission (*n*,%)	116 (8.8)	32 (26.2)	84 (7.1)	** < 0.001**
Noninvasive ventilation (*n*,%)	365 (27.8)	59 (48.4)	306 (25.7)	** < 0.001**
Invasive ventilation (*n*,%)	248 (18.9)	37 (30.3)	211 (17.7)	**0.001**
*Complications during hospitalization (n,%)*				
Respiratory failure	323 (24.6)	50 (41.0)	273 (22.9)	** < 0.001**
Heart failure	327 (24.9)	42 (34.4)	285 (23.9)	**0.011**
Nosocomial pneumonia	109 (8.3)	21 (17.2)	88 (7.4)	** < 0.001**
Septic shock	119 (9.1)	24 (19.7)	95 (8.0)	** < 0.001**
Nosocomial BSI	18 (1.4)	9 (7.4)	9 (0.8)	** < 0.001**
Acute renal failure	79 (0.6)	8 (6.6)	71 (6.0)	0.592
Admittance to ICU (*n*,%)	326 (24.8)	59 (48.4)	267 (22.4)	** < 0.001**
Days from clinical stability to admission (median, IQR)	3.0 (1.0–9.0)	14.0 (10.0–19.0)	3.0 (1.0–8.0)	** < 0.001**
Length of stay in hospital (days, median, IQR)	10.0 (8.0–17.0)	17.0 (11.0–22.0)	10.0 (8.0–14.0)	** < 0.001**
30-day mortality (*n*,%)	315 (24.0)	46 (37.7)	242 (20.3)	** < 0.001**

*IC* immunocompromised, *IQR* interquartile range, *SD* standard deviation, *COPD* chronic obstructive pulmonary disease, *SBP* systolic blood pressure, *HB* hemoglobin, *ALB* albumin, *BUN* blood urea nitrogen, *PaO*_*2*_*/FiO*_*2*_ arterial pressure of oxygen/fraction of inspiration oxygen, *NAI* neuraminidase inhibitor, *BSI *bloodstream infection, *ICU* The bolded values are p-values < 0.05, which represented significant differences between IC and non-IC patients with Flu-p. The bolded values are p-values < 0.05, which represented significant differences between IC and non-IC patients

Compared to non-IC patients, IC patients (43.4% *vs.* 34.0%, p = 0.037) were more commonly coinfected with other pathogens at admission. Incidence of gram-negative bacterium (69.8% *vs.* 48.4%, p = 0.003), fungus (11.3% *vs.* 0.5%, p < 0.001) and *Cytomegalovirus* (CMV) (3.8% *vs.* 0.0%, p < 0.001) was higher in IC patients, while the proportion of gram-positive bacterium (37.7% *vs.* 55.1%, p = 0.017) was lower (Additional file 1: Material S5).

All of the Flu-p patients were administered antibiotics during their hospital stays. Compared to non-IC patients, less IC patients were administrated with systemic corticosteroids at admission (7.1% *vs* 26.2%, p < 0.001); more IC patients received noninvasive (48.8% *vs.* 25.7%, p < 0.001) and invasive (30.3% *vs.* 17.7%, p = 0.001) ventilation. Additionally, respiratory failure (41.0% *vs.* 22.9%, p < 0.001), heart failure (34.4% *vs.* 23.9%, p = 0.011), nosocomial pneumonia (17.2% *vs.* 7.4%, p < 0.001), septic shock (19.7% *vs.* 8.0%, p < 0.001) and nosocomial blood stream infections (7.4% *vs.* 0.8%, p < 0.001) were more frequent in IC patients. The duration between admission to clinical stability (median: 14.0 days *vs.* 3.0 days, p < 0.001), as well as the length of hospital stays (median: 17.0 days *vs.* 10.0 days, p < 0.001) were significantly longer for IC patients than for non-IC patients. Finally, more IC patients were admitted to the ICU (48.4% *vs.* 22.4%, p < 0.001), and the all-cause 30-day mortality rate (37.7% *vs.* 20.3%, p < 0.001) was higher in IC patients (Table 1).

### Impact of immunocompromised status on clinical outcomes for Flu-p patients

After controlling for age, sex, duration between illness onset and admission, influenza virus type, comorbidities, pregnancy, obesity, smoking history, early NAI therapy, administration of systemic corticosteroids, antibiotics use, and coinfection with other community-acquired pathogens, IC status was associated with increased risks for invasive ventilation [*odds ratio* (*OR*): 2.475, *95% confidence interval* (*IC*): 1.511–4.053, p < 0.001], ICU admission (*O:R* 3.247, *95% CI* 2.064–5.106, p < 0.001) and 30-day mortality (*OR:* 3.206, *95% CI* 1.926–5.335, p < 0.001) for patients with Flu-p (Table 2).

**Table 2:** The impact of immuncompromised status on the clinical outcomes of Flu-p patients

Clinical outcomes	Univariate logistic analysis		Multivariate logistic analysis	
	***OR* (*95% CI*)**	***P* value**	***OR* (*95% CI*)**	***p* value**
Invasive ventilation	2.022 (1.337–3.058)	0.001	2.475 (1.511–4.053)	< 0.001
ICU admission	3.241 (2.216–4.741)	< 0.001	3.247 (2.064–5.106)	< 0.001
30-day mortality	2.374 (1.603–3.514)	< 0.001	3.206 (1.926–5.335)	< 0.001

*OR* odd ratio, *CI* confidence interval

### Baseline predictors for 30-day mortality in IC patients with Flu-p

Results from univariate analysis revealed that respiratory rates ≥ 30 breaths/min at admission, and decreased baseline blood lymphocytes, blood albumin, PaO_2_/FiO_2_ and coinfection were associated with an increased risk for 30-day mortality in IC patients with Flu-p (Additional file 1: Material S6).

A multivariate backward stepwise logistic regression suggested that lymphocyte counts (*OR:* 0.993*, 95% CI* 0.990–0.996, p < 0.001), coinfection (*OR:* 5.450*, 95% CI* 1.638–18.167, p = 0.006), early NAI therapy (*OR:* 0.401*, 95% CI* 0.127–0.878, p = 0.001), and systemic corticosteroid use at admission (*OR:* 6.414*, 95% CI* 1.348–30.512, p = 0.020) were independent predictors for 30-day mortality in IC patients with Flu-p (Table 3).

**Table 3:** Risk factors for mortality in IC patients with Flu-p

Variable	*OR* (*95% CI*)	*p* value
Lymphocyte counts	0.993 (0.990–0.996)	< 0.001
Coinfection	5.450 (1.638–18.167)	0.006
Early NAI therapy	0.401 (0.127–0.878)	0.001
Systemic corticosteroids use	6.414 (1.348–30.512)	0.020

### Association of coinfected pathogens with baseline lymphocytes and 30-day mortality in IC patients with Flu-p

**Fig. 2:**
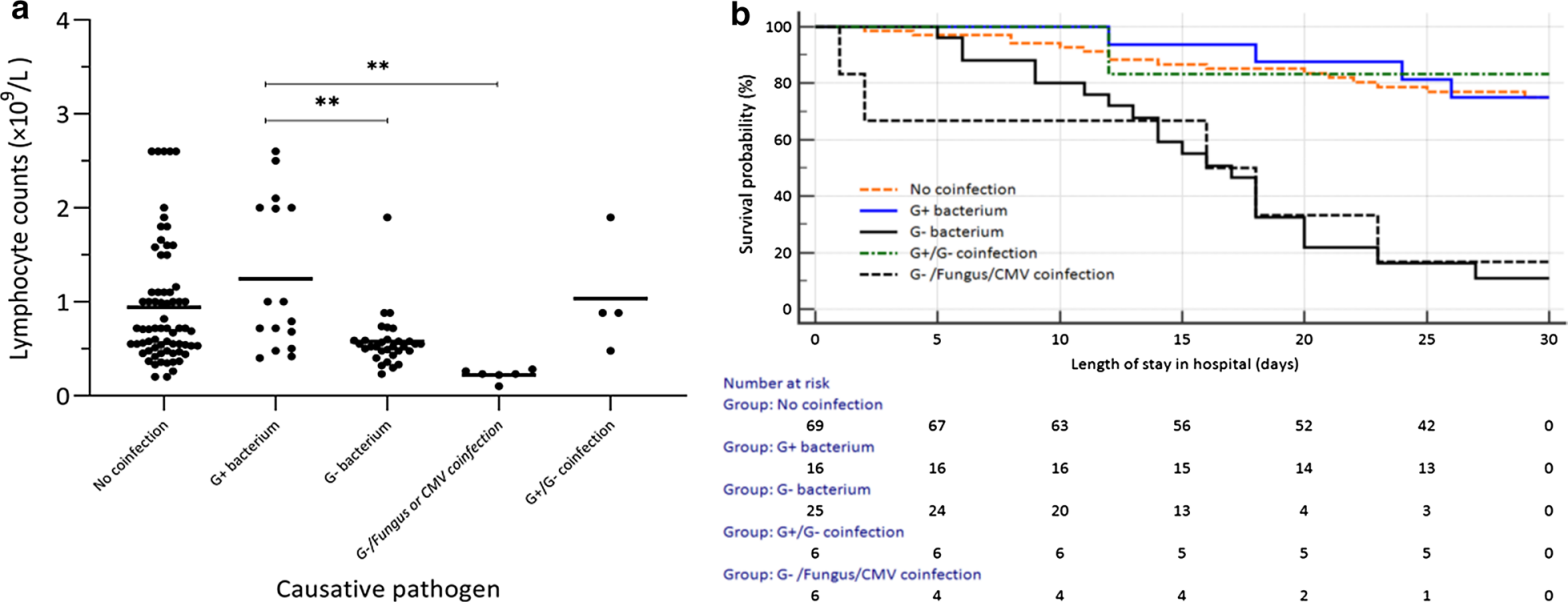
**A** Comparison of lymphocyte counts of patients with different causative pathogens; **B** Kaplan–Meier curves showing the survival probabilities of patients with different causative pathogens. (**: p < 0.001). **A** The baseline lymphocyte count levels for IC patients coinfected with gram-positive bacterium was significantly higher than those for IC patients coinfected with gram-negative bacterium, gram-negative bacterium/fungus or CMV; **B** The survival rates of IC patients coinfected with gram-negative bacteriumand gram-negative bacterium/fungus or CMV were lower than those of IC patients coinfected with gram-positive bacterium during the first 30 days after admission

The median baseline lymphocyte count levels for IC patients coinfected with gram-positive bacterium (0.9 × 10^9^/L) was significantly higher than those for IC patients coinfected with gram-negative bacterium (0.5 × 10^9^/L, p < 0.001), gram-negative bacterium/fungus or CMV (0.2 × 10^9^/L, p < 0.001) (Fig. 2A).

During the first 30 days after admission, survival rates of IC patients coinfected with gram-negative bacteria (80.0% *vs.* 25.0%, p < 0.001), gram-negative bacteria and fungus, or CMV (83.3% *vs.* 25.0%, p < 0.001) were lower than those of IC patients coinfected with gram-positive bacterium (25.0%) (Fig. 2B).

### Baseline lymphocytes as predictors for 30-day mortality in IC patients with Flu-p

**Fig. 3:**
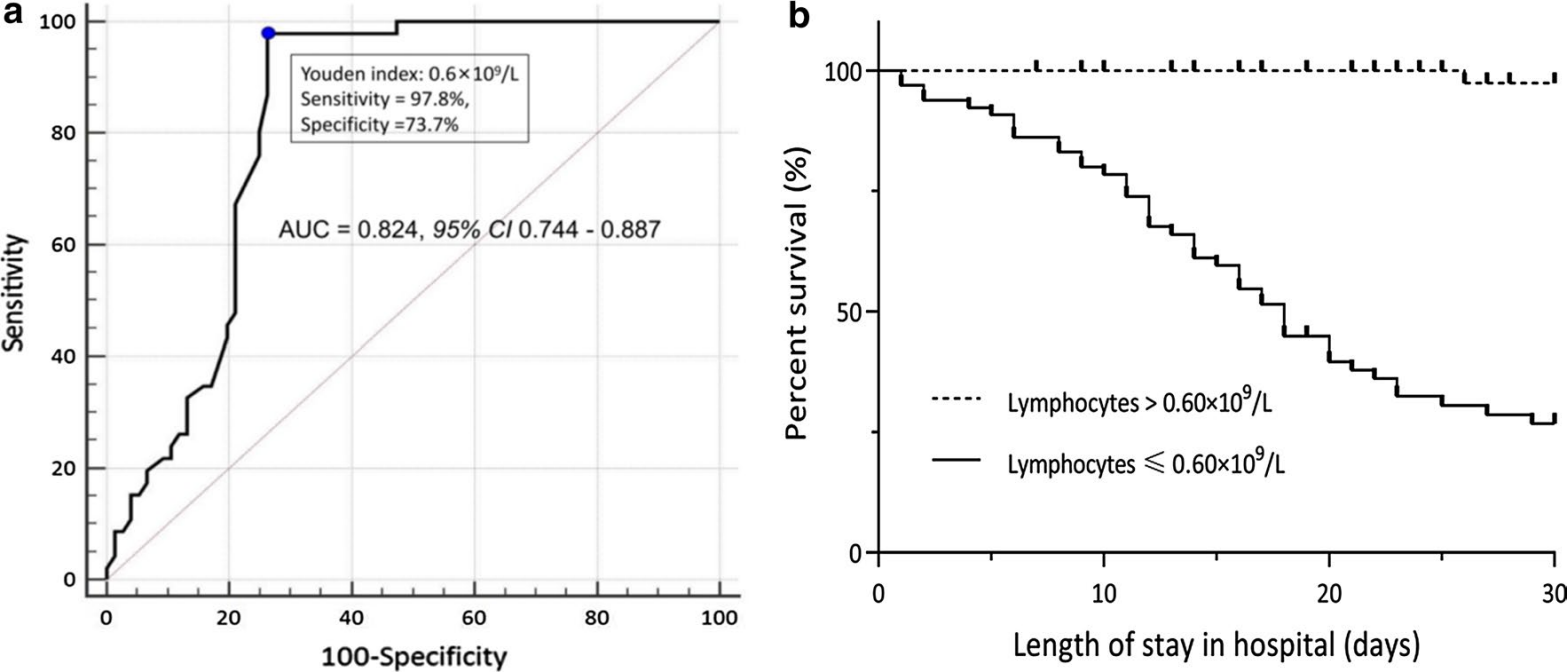
**A** ROCs for mortality prediction of lymphocytes among IC patients with Flu-p; **B** Kaplan–Meier curves showing the survival probabilities of patients with two levels of lymphocyte counts. **A** The ROC determined that the optimal cutoff of baseline lymphocyte counts was 0.6 × 10^9^/L, which reached an AUROC of 0.825 (*95% CI* 0.744—0.887), with sensitivity of 97.8% and specificity of 73.7%; **B** The Kaplan–Meier curves showed that the 30-day mortality of IC patients with baseline lymphocyte counts ≤ 0.6 × 10^9^/L was higher than that of patients with lymphocyte counts > 0.6 × 10^9^/L

The ROC determined that the optimal cutoff of baseline lymphocyte counts was 0.6 × 10^9^/L, which reached an AUROC of 0.825 (*95% CI* 0.744–0.887), with sensitivity of 97.8% and specificity of 73.7% (Fig. 3A).

The Kaplan–Meier curves showed that the 30-day mortality of IC patients with baseline lymphocyte counts ≤ 0.6 × 10^9^/L was higher than that of patients with lymphocyte counts > 0.6 × 10^9^/L (69.2% *vs.* 1.8%, log-rank test, p < 0.001) (Fig. 3B).

## Discussion

In this multicenter study, we found that IC status correlated with worse clinical outcomes in patients hospitalized with Flu-p. Additionally, we identified several early predictors of mortality for IC patients with Flu-p, which may help inform treatment regimens.

In our cohort, IC patients accounted for 9.3% of all Flu-p patients, which was consistent with previous reports of 5–15% IC incidence in severe influenza [5–10]. Compared to non-IC patients with Flu-p, we found that IC patients tended to be younger, presented with chest pain and pleural effusion more often, presented with fevers less frequently, and had lower level of blood lymphocytes. The other baseline clinical features were similar. Memoli and colleagues [15] reported that IC patients with influenza demonstrated fewer clinical symptoms and signs on clinical assessment. However, Collins et al. [16] showed that some symptoms and signs (e.g., fever, sore throat, myalgias, and headaches) were more common in IC patients with influenza. One possible explanation was for this difference is the discrepancy in evaluation timing. As influenza is a potentially progressive disease, some clinical features might be latent early in the disease course [17]. Another potential cause is that prior use of systemic corticosteroids and immunosuppressants might conceal symptoms in IC patients with influenza [18, 19].

Although similar baseline clinical characteristics were observed between the patients with and without immunocompromising conditions, disease severity in IC patients was greater than that in immunocompetent patients. IC adults were more likely to experience worse outcomes, including longer hospital stays, more complications, increased risks for intubation, and increased risk for ICU admission and death, all of which were in line with most previous reports [5–10, 15, 16]. However, we used a more broad definition of immunocompromised status, which means our results have more general significance.

We also identified risk factors that were predictors for death in IC patients with Flu-p, many of which have also been related to outcomes among immunocompetent influenza patients in previous research. Severe influenza is characterized by lymphocytopenia in 30–100% of cases [20, 21], although the mechanistic basis for this finding remains poorly understood. There is some evidence that CD4 + and CD8 + T cells may undergo higher rates of apoptotic death in individuals with severe disease, owing to higher circulating levels of soluble Fas ligand and caspase-1 [22], and thereby contributing to an overall decline in lymphocyte counts. Such virus-induced lymphocytopenia can delay viral clearance. Alternatively, these lymphocytes may be recruited to the respiratory tract and other organs, resulting in their apparent depletion from circulation [23]. Lymphocyte accumulation within the lungs can drive severe localized inflammation and tissue damage. Previous studies reported that the predictive value of baseline blood lymphocyte counts was 0.8 × 10^9^/L in immunocompetent patients with severe influenza [24, 25]. However, because individuals with compromised factors often have low lymphocyte counts before coming down with influenza [26, 27], it is reasonable to expect that the optimal cutoff could differ. Su-Mi Choi et al. reported that, in hematopoietic cell transplant recipients with influenza, profound lymphopenia (< 300 × 10^9^/L) was a significant risk factor for death [28]. In HIV/ARDS individuals with A (H1N1) infection, patients with CD4 T-cell count < 200 × 10^9^/L experienced worse outcomes [29]. Our study suggested that, in general, in IC patients with Flu-p, baseline lymphocyte counts of 0.6 × 10^9^/L could be used as an independent prognostic factor.

Coinfection has previously been shown to be related to adverse outcomes in immunocompetent patients with severe influenza [30, 31], as well as in IC individuals, highlighting the importance of timely infection control. Further, we found the survival rates of IC patients coinfected with gram-negative bacteria or fungus/CMV were significantly lower than those coinfected with gram-positive pathogens. Additionally, IC patients coinfected with gram-negative bacteria often had lower baseline blood lymphocyte counts. Thus, lymphopenia might be associated with increased mortality among Flu-p patients in multiple ways.

Previous observational studies have suggested that inhibiting viral replication at an early stage can reduce virus-induced inflammation and tissue damage, and thereby decrease overall influenza-related mortality rates for immunocompetent patients [32]. Our results showed it was still associated with better outcomes in IC patients with Flu-p. Early inhibition of viral replication might be particularly crucial in IC patients with influenza, because prolonged viral shedding is associated with the emergence of drug-resistant viruses and more severe disease courses [33, 34]. Meanwhile, systemic corticosteroids use may cause increased death by extensive suppression of humoral and cellular immunity [35], even in patients with immunocompromised factors.

Our study design has some limitations. First, as it was retrospective in nature, it was susceptible to selection bias. For example, nucleic acid tests for influenza were conducted based upon the subjective judgment of attending physicians. Thus, it is possible that only patients with more severe disease manifestations may have undergone testing, rather than all potentially eligible patients. In addition, previous reports have suggested that empiric antibiotic therapy is important for pneumonia patients [36]. In our study, although only 34% of patients were coinfected with other pathogens, all patients were given antibiotics. Thus, we were unable to assess the impact of proper empiric antibiotic treatment on Flu-p patient outcomes. Second, the sample of IC patients was relatively small. Third, because of the retrospective study design, we were unable to retrieve and evaluate vaccination data or other missing information, potentially constraining the accuracy of our results.

Despite these limitations, however, our study showed that IC status has a consistent and wide-ranging negative impact on clinical outcomes among patients hospitalized with Flu-p. The risk factors for mortality identified in our study may be helpful for clinicians who manage IC patients with Flu-p.

## Supplementary Information

**Additional file 1: **Supplementary data of this article.

## Data Availability

The datasets used and/or analysed during the current study are available from the corresponding author on reasonable request.

## Abbreviations

Flu: Influenza
Flu-p: Influenza-related pneumonia
IC: Immunocompromised
ICU: Intensive care unit
NAI: Neuraminidase inhibitor
HIV/ARDS: Human immunodeficiency virus/acquired immune deficiency syndrome
OR: Odds ratio
95% CI: 95% Confidence interval
AUROC: Under the receiver-operating characteristic curve
ICU: Intensive care unit
