# Supplementary material for: The severity and risk factors for mortality in immunocompromised adult patients hospitalized with influenza-related pneumonia
Source: Ann Clin Microbiol Antimicrob. 2021 Aug 24;20:55. doi: 10.1186/s12941-021-00462-7 (PMC8383249; doi:10.1186/s12941-021-00462-7)
Supplement: Supplementary file 1 — Additional file 1: Supplementary data of this article. [file 12941_2021_462_MOESM1_ESM.docx]

**Material S1: Details of participating centers**

| **Name of the hospital** | **Province, city** | **Teaching Hospital** | **Beds** | **Staffs of Clinical Microbioloy Lab** |
| --- | --- | --- | --- | --- |
| Beijing Jishuitan Hospital, | Beijing | Yes | 1500 | 10 |
| Beijing Chao-Yang Hospital | Beijing | Yes | 1400 | 11 |
| **the 2^nd^ People’s Hospital of Yunnan Province** | **Kunming,**  **Yan’an** | Yes | 1302 | 4 |
| **Qingdao Municipal Hospital** | ShanDong,  Qingdao | Yes | 1200 | 4 |
| Beijing Huimin Hospital | Beijing | Yes | 1000 | 2 |

**Material S2 Definition of underlying diseases**

1. Smoking was defined as cigarette smokers of 10 cigarettes/d during at least the previous year；
2. Cardiovascular disease included coronary heart disease and chronic congestive heart failure;;
3. Coronary heart disease included angina pectoris, myocardial infarction, ischemic cardiomyopathy;
4. Heart failure was defined as a clinical syndrome consisting of dyspnea, malaise, swelling and/or decreased exercise capacity due to the loss of compensation for cardiac pumping function due to structural and/or functional abnormalities of the heart;
5. Cerebrovascular diseases included transient ischemic attack, cerebral hemorrhage, subarachnoid hemorrhage, cerebral infarction;
6. Diabetes mellitus: included diabetes mellitus type 1 and diabetes mellitus type 2, not included impaired glucose tolerance and impaired fasting glycaemia;
7. Chronic obstructive pulmonary disease was defined as: persistent airflow limitation, FEV_1_ / FVC < 70% post bronchodilator;
8. Asthma was defined by the history of respiratory symptoms such as wheeze, cough that varied over time and intensity, together with variable respiratory airway limitation;
9. Chronic kidney disease included diabetic nephropathy, hypertensive renal damage, chronic glomerulonephritis, chronic pyelonephritis, lupus nephritis, IgA nephropathy, nephrotic syndrome, hereditary kidney disease;
10. Obesity was defined as body mass index (BMI) ≥ 30 kg/m^2^;
11. Immunosuppressive therapy: was defined as receiving cancer chemotherapy, receiving corticosteroid therapy with a dose ≥ 20 mg prednisone or equivalent daily for ≥ 14 d or a cumulative dose > 600 mg of prednisone, receiving biological immune modulators, receiving disease-modifying antirheumatic drugs or other immunosuppressive drugs (eg, cyclosporin, cyclophosphamide, hydroxychloroquine, methotrexate).
12. Mental confusion was defined as a mental test score of 8 or less or disorientation in person, place or time.

**Material S3 Definition of microbiological criteria of coinfected with other pathogens**

1. Positive urinary antigen for *Legionella pneumophila*;
2. Positive urinary antigen for *Streptococcus pneumoniae*;
3. Positive bacterial culture from blood or plural fluid except for coagulase negative *Staphylococcus spp*.;
4. Paired sera with a fourfold or more increase in the titers of antibodies to *Mycoplasma pneumoniae* (MP), *Chlamydia pneumonia*, *L pneumophila or* respiratory viruses ( Parainfluenza, Adenovirus, Respiratory syncytial virus)*.* Or Serum IgM antibody (MIF) ≥ 1:16 for *Chlamydia pneumonia*;
5. Detection of respiratory virus in sputum/bronchoalveolar lavage (BALF)/throat swabs by Realtime-PCR according to manufacturer’s instructions, including respiratory syncytial virus (RSV) types A and B, parainfluenza virus (PIV) types 1, 2, 3 and 4, rhinovirus (HRV), enterovirus (EV), coronavirus (hCoV) types 229E, NL63, OC43 and HKU1, parapneumovirus (hMPV), and adenovirus (AdV), bocavirus;
6. Bacteria isolated form purulent sputum (defined as an adequate quality sputum sample with > 25 leukocytes and < 10 epithelial cells per × 100 magnification field) with compatible findings of Gram staining;
7. Detection of *Mycoplasma pneumoniae* (MP), *Chlamydia pneumonia* or *L pneumophila* in sputum/BALF/throat swabs by Realtime-PCR;
8. serum IgM antibody positive for *Mycoplasma pneumoniae* (MP), or Serum IgG antibody (MIF) ≥ 1:512 for *Chlamydia pneumonia;*
9. Invasive pulmonary aspergillosis were diagnosed in accordance with the revised definitions of invasive fungal diseases from the European Organization for Research and Treatment of Cancer and the Mycoses Study Group Education and Research Consortium [1].
10. Cytomegalovirus (CMV) pneumonia was defined by the presence of signs and/or symptoms of pulmonary disease combined with the detection of CMV in bronchoalveolar lavage fluid or lung tissue samples. Detection of CMV should be performed by virus isolation, histopathologic testing, immunohistochemical analysis, or in situhybridization [2].
11. Pneumocystis jirovecii (PJP) diagnosis was based on a positive direct microscopy by immunofluorescence on induced sputum or bronchoalveolar lavage (BAL) and/or a positive PCR assay on a BAL specimen [3].

References

1. Donnelly JP, Chen SC, Kauffman CA, et al. Revision and Update of the Consensus Definitions of Invasive Fungal Disease From the European Organization for Research and Treatment of Cancer and the Mycoses Study Group Education and Research Consortium. Clin Infect Dis. 2019, pii: ciz1008.
2. Per Ljungman, Paul Griffiths, Carlos Paya. Definitions of cytomegalovirus infection and disease in transplant recipients. Clin Infect Dis. 2002;34(8):1094-7
3. Alanio A, Hauser PM, Lagrou K, Melchers WJ, Helweg-Larsen J, Matos O, et al. ECIL guidelines for the diagnosis of Pneumocystis jirovecii pneumonia in patients with haematological malignancies and stem cell transplant recipients. J Antimicrob Chemother. 2016;71(9):2386-96.

**Material S4 Underlying immunocompromising conditions of the IC patients with Flu-p**

| **Immunocompromised factor** | **Cases (*n*, %)** |
| --- | --- |
| Immunosuppressive therapy | 57 (46.7) |
| Active malignancy (no transplant) | 25 (20.5) |
| Organ transplantation | 17 (13.9) |
| Stem-cell transplant | 13 (10.7) |
| HIV(+)/AIDS | 6 (4.9) |
| Primary immune deficiency diseases | 2 (1.6) |
| Splenectomy | 2 (1.6) |

**Material S5 coinfections with other community-acquired pathogens**

| **Variable** | **IC Flu-p (*n* = 122)** | **Non-IC Flu-p (*n* = 1191)** | ***p* value** |
| --- | --- | --- | --- |
| Coinfection (*n*, %) | 53 (43.4) | 405 (34.0) | 0.037 |
| Pathogens (*n*, %) |  |  |  |
| Gram postive | 20 (37.7) | 223 (55.1) | 0.017 |
| *Streptococcus pneumoniae* | 5 (9.4) | 121 (29.9) |  |
| *Staphylococcus aureus* | 13 (24.5) | 84 (20.7) |  |
| *Other streptococcus spp.* | 2 (3.8) | 18 (4.4) |  |
| Gram negative | 37 (69.8) | 196 (48.4) | 0.003 |
| *Klebsiella pneumoniae* | 16 (30.2) | 133 (32.8) |  |
| *Haemophilus influenzae* | 0 (0.0) | 31 (7.7) |  |
| *Klebsiella acidogens* | 0 (0.0) | 9 (2.2) |  |
| *Escherichia coli* | 4 (7.5) | 6 (1.5) |  |
| *Pseudomonas aeruginosa* | 8 (15.1) | 5 (1.2) |  |
| *Proteus spp.* | 3 (5.7) | 4 (1.0) |  |
| *Citrobacter spp.* | 0 (0.0) | 3 (0.7) |  |
| *Flavobacterium* | 0 (0.0) | 2 (0.5) |  |
| *Acinetobacter* | 3 (5.7) | 2 (0.3) |  |
| *Stenotrophomonas maltophilia* | 3 (5.7) | 1 (0.2) |  |
| Fungus | 6 (11.3) | 2 (0.5) | < 0.001 |
| *Aspergillus spp.* | 2 (3.8) | 2 (0.5) |  |
| *Pneumocystis jirovecii* | 4 (7.5) | 0 (0.0) |  |
| *Cytomegalovirus* | 2 (3.8) | 0 (0.0) | < 0.001 |

12 IC patients and 16 non-IC patients were coinfected with 2 or more pathogens

**Material S6 Comparison of clinical characteritics and treatments between deceased and survival IC patients with Flu-p**

| **Variable** | **Deaceased IC patients**  **(*n* = 46)** | **Survival IC patients**  **(*n* = 76)** | ***p* value** |
| --- | --- | --- | --- |
| Age (yrs, mean ± SD) | 42.3 ± 15.6 | 43.4 ± 14.1 | 0.287 |
| Male (*n*, %) | 22 (47.8) | 39 (51.3) | 0.709 |
| Influenza A infection (*n*, %) | 10 (21.7) | 25 (32.9) | 0.187 |
| Comorbidities (*n*, %) |  |  |  |
| Cardiovascular disease | 10 (21.7) | 12 (15.8) | 0.407 |
| Cerebrovascular disease | 7 (15.2) | 7 (9.2) | 0.313 |
| Diabetes mellitus | 6 (13.0) | 8 (10.5) | 0.672 |
| COPD | 6 (13.0) | 9 (11.8) | 0.845 |
| Asthma | 1 (2.2) | 4 (5.3) | 0.717 |
| Chronic kidney disease | 3 (6.5) | 4 (5.3) | 1.000 |
| Obesity (*n*, %) | 2 (4.3) | 3 (3.9) | 1.000 |
| Smoking history (*n*, %) | 12 (26.1) | 15 (19.7) | 0.413 |
| Baseline clinical and radiologic features |  |  |  |
| Respiratory rates ≥ 30 breaths/min ^#^(*n*, %) | 15 (32.6) | 4 (5.3) | **< 0.001** |
| Altered mental status (*n*, %) | 4 (8.7) | 8 (10.5) | 0.988 |
| SBP < 90 mmHg (*n*, %) | 2 (4.3) | 2 (2.6) | 1.000 |
| Leukocytes (×10^9^/L, median, IQR) | 6.3 (3.4, 11.9) | 6.3 (4.6, 8.9) | 0.799 |
| Lymphocytes ^#^ (×10^9^/L, median, IQR) | 0.5 (0.4, 0.6) | 0.9 (0.6, 1.6) | **< 0.001** |
| HB (g/L, mean ± SD) | 121.2 ± 27.2 | 123.9 ± 20.5 | 0.540 |
| ALB ^#^ (g/L, mean ± SD) | 26.0 ± 4.9 | 29.9 ± 6.2 | **< 0.001** |
| BUN (mmol/L, median, IQR) | 7.7 (3.3, 8.4) | 4.9 (3.1, 8.1) | 0.435 |
| PaO_2_/FiO_2_ ^#^ (mmHg, median, IQR) | 312.5 (264.3, 323.3) | 325.2 (277.4, 352.4) | **0.005** |
| Multilobar infiltrates (*n*, %) | 32 (69.6) | 53 (69.7) | 0.984 |
| Pleural effusion (*n*, %) | 22 (47.8) | 36 (47.4) | 0.961 |
| Coinfection ^#^ (*n*, %) | 30 (65.2) | 23 (30.3) | **< 0.001** |
| Early NAI therapy ^#^ (*n*, %) | 16 (34.8) | 39 (51.3) | 0.075 |
| Systemic corticosteroids use at admission ^#^ (*n*, %) | 16 (34.8) | 16 (21.1) | 0.095 |

IC: immunocompromised; SD: standard deviation; COPD: chronic obstructive pulmonary disease; SBP: systolic blood pressure; HB: hemoglobin; ALB: albumin; BUN: blood urea nitrogen; PaO_2_/FiO_2_: arterial pressure of oxygen/fraction of inspiration oxygen; NAI: neuraminidase inhibitor. ^#^: variables cited in the table above were the candidates which were entered into the multivariate logistic regression model. The bolded values are p-values < 0.05, which represented significant differences between deceased and survival patients.
